# Mesenchymal stromal cells in the bone marrow niche consist of multi-populations with distinct transcriptional and epigenetic properties

**DOI:** 10.1038/s41598-021-94186-5

**Published:** 2021-08-04

**Authors:** Sanshiro Kanazawa, Hiroyuki Okada, Hironori Hojo, Shinsuke Ohba, Junichi Iwata, Makoto Komura, Atsuhiko Hikita, Kazuto Hoshi

**Affiliations:** 1 Department of Oral and Maxillofacial Surgery, Graduate School of Medicine, The University of Tokyo, Tokyo, Japan; 2 Center for Disease Biology and Integrative Medicine, Graduate School of Medicine, The University of Tokyo, Tokyo, Japan; 3 Department of Bioengineering, Facility of Medicine, The University of Tokyo, Tokyo, Japan; 4 Department of Cell Biology, Medical and Dental Sciences, Graduate School of Biomedical Sciences, Nagasaki University, Nagasaki, Japan; 5 Department of Diagnostic and Biomedical Sciences, The University of Texas Health Science Center At Houston, Houston, TX USA; 6 Division of Tissue Engineering, The University of Tokyo Hospital, Tokyo, Japan; 7 Department of Cell and Tissue Engineering (Fujisoft), Graduate School of Medicine, The University of Tokyo, Tokyo, Japan

**Keywords:** Stem cells, Mesenchymal stem cells

## Abstract

Although multiple studies have investigated the mesenchymal stem and progenitor cells (MSCs) that give rise to mature bone marrow, high heterogeneity in their morphologies and properties causes difficulties in molecular separation of their distinct populations. In this study, by taking advantage of the resolution of the single cell transcriptome, we analyzed Sca-1 and PDGFR-α fraction in the mouse bone marrow tissue. The single cell transcriptome enabled us to further classify the population into seven populations according to their gene expression profiles. We then separately obtained the seven populations based on candidate marker genes, and specified their gene expression properties and epigenetic landscape by ATAC-seq. Our findings will enable to elucidate the stem cell niche signal in the bone marrow microenvironment, reconstitute bone marrow in vitro, and shed light on the potentially important role of identified subpopulation in various clinical applications to the treatment of bone- and bone marrow-related diseases.

## Introduction

The bone marrow microenvironment, which is a multifunctional network between cells and the extracellular matrix, plays crucial roles in maintenance of proliferation, differentiation, and survival of stromal cells and hematopoietic cells^1^. One important cell lineage derived from bone marrow is mesenchymal stem/progenitor cells (MSCs). These cells have the ability to differentiate into various lineage such as osteocytes, chondrocytes, adipocytes, etc., corresponding to external signals from the microenvironment and endogenous ones inducing commitment of cell lineages.

Among various kinds of cells in the bone marrow, hematopoietic stem cells (HSCs) have been intensively studied. The research field has been progressed because markers for isolating HSCs have been identified. CD34-/KLS and CD34-KLS/CD150+ have been defined as markers for HSCs by international society for stem cell research^2,3^. About 1000–3000 HSCs can be harvested per mouse at almost 100% purity, while differentiation and cell death taking place during collection decrease the purity of HSCs about 20–50%^4^. Many studies are also conducted on MSCs^5–8^. Several combinations of markers have been discovered for isolating MSCs, and functions of the isolated cells were evaluated^9–12^. However, cells expressing these markers are still composed of heterogeneous cell populations, which hinders precise characterization of MSCs^13–21^. For example, Sca-1 (Ly6 A/E) and PDGFR-α (Pα-S) are currently considered to be useful for the MSCs separation^22^. However, the cells separated using these markers contain heterogenous progenitor populations, within which bona-fide MSCs exist because part of these cells express Nestin, NG2 or leptin receptor in vivo^23^, which is considered as a MSC marker^24^. In addition, pericytes expressing Tbx18 are also positive for MSC marker such as CD146 or Sca-1, indicating the heterogeneity of Pα-S population^15^. To understand the nature of MSCs more profoundly, it is quite important to dissect the heterogenous populations of Pα-S, and analyze each population separately.

In this study, we identified subpopulations in mouse bone marrow MSCs positive for Pα-S by single-cell level with single-cell RNA-seq (scRNA-seq) analysis. Assay for Transposase Accessible Chromatin with sequencing (ATAC-seq) analysis of each of the subpopulations further revealed epigenetic landscape, their genetic similarities and functional properties. Our findings will contribute to the elucidation of the essential characteristics of MSCs, which lead to the safe and effective application of the cells to clinical settings.

## Results

### Identification of heterogenic cell population in isolated MSCs using scRNA-seq

**Figure 1:**
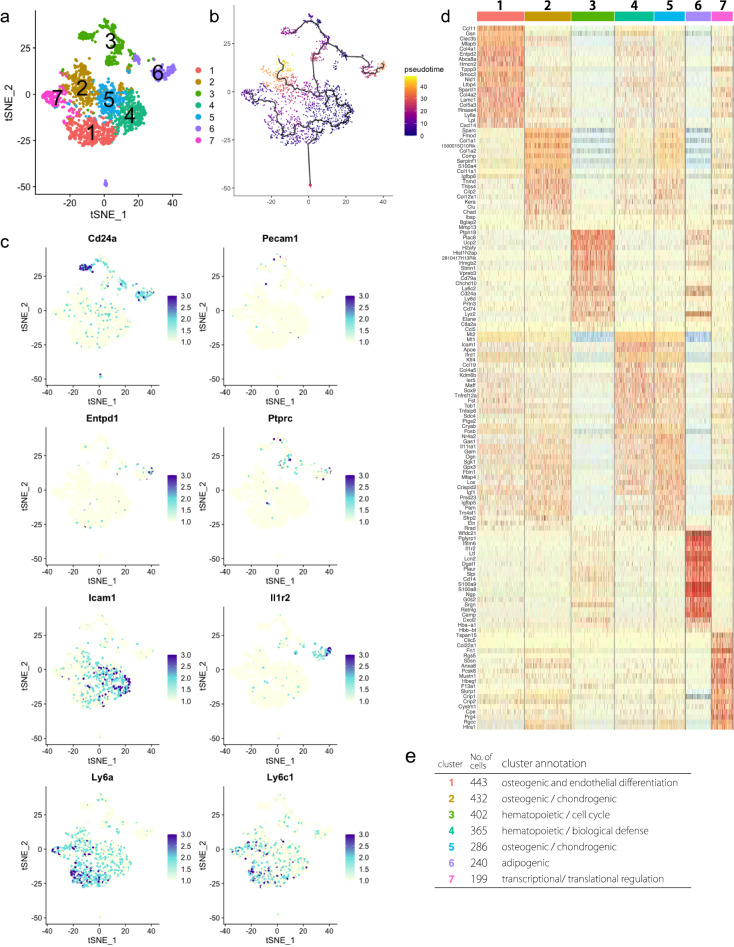
Single cell RNA-seq (scRNA-seq) analysis for the Pα-S fraction cells (n = 2367). (**a**) Dimension reduction map with tSNE by Cell Ranger. (**b**) Pseudotime analysis by Monocle3 on seurat object, setting origin to cluster 4. (**c**) Feature plot for marker genes, Cd24a, Cd31 (Pecam-1), Cd39 (Entpd1), Cd45 (Ptprc), Cd54 (Icam1), Cd121b (Il1r2), Sca-1 (Ly6a), Ly6c1. (**d**) Heatmap of top 10 upregulated genes in each cluster (color code as in Fig. 1a). (**e**) Table of each cluster annotation in functional features.

Currently, selection markers useful for isolating bone marrow-derived stem/ progenitor cells (MSCs) with advances in FACS technology dramatically improve purification of the cell population compared to conventional methods^25^. As a consequence, the analysis of MSC’s properties had significantly progressed, and the immunosuppression^18^ and anti-inflammatory capacity of MSCs with multipotency have been shown^26,27^. However, since more than half of the cells isolated as MSCs are heterogenic cell population, it remains unknown whether they are the main function of MSCs. Therefore, it is important to clarify what kind of cell population the isolated MSCs have. To answer these questions, we first performed scRNA-seq using a platform of Chromium Controller (10× Genomics, Inc) in 30,000 of MSCs^28^, which were negative for CD31, 45, and Ter119 and positive for Sca-1 and Pdgfr-α (Fig. S1, a). Based on this distribution for Cell Ranger, all 2367 cells that could be analyzed were targeted without cutoff (Fig. S1, b and c). The similarity of gene expression status between cells was evaluated by tSNE analysis, and the diversity of cells was visualized. As a result, we found that the seven different cell subpopulations exist in isolated MSCs according to genetic similarities (Fig. 1a). We suggested that the subpopulation in the Pα-S fraction present diversity in the differentiation state. Therefore, we performed pseudotime analysis to visualize the differentiation state of each population by associating clusters with analogy of gene expression states using Monocle 3. As a result, we confirmed that the differentiation state altered around cluster 4 (Fig. 1b). We also examined for cell surface markers that are effective for FACS isolation of clusters. Especially, Cd24a, Cd31 (Pecam-1), Cd39 (Entpd1), Cd45 (Ptprc), Cd54 (Icam1), Cd121b (Il1r2), Sca-1 (Ly6a) and Ly6c1 were focused as up- and down-regulated marker genes in comprehensive examination of cell markers (Fig. 1c and Fig. S1, d). Annotation analysis from top 10 upregulated genes in each cluster in the gene expression profile suggest that cluster 1 may have osteogenic and endothelial differentiation properties, clusters 2 and 5 may have osteogenic/ chondrogenic ones, cluster 6 may have adipogenic directionality, cluster 7 may have angiogenic properties as shown by the upregulated expression of Rgcc and Rgs5, and clusters 3 and 4 showed characteristic of the hematopoietic lineage (Fig. 1d). Furthermore, we found functional features of the clusters based on expressed genes: cluster 1, cell aging; cluster 3, cell cycle; cluster 4, biological defense; and cluster 7, transcriptional/ translational regulation (Fig. 1e). We performed normalization, dimensionality reduction and clustering using Seurat from the gene expression profile of scRNA-seq, and implemented down-stream analyses of scRNA-seq for the Pα-S fraction cells (n = 2367) such as visualization of trajectory depiction and GO analysis. We compared the enriched pathways between clusters.

**Figure 2:**
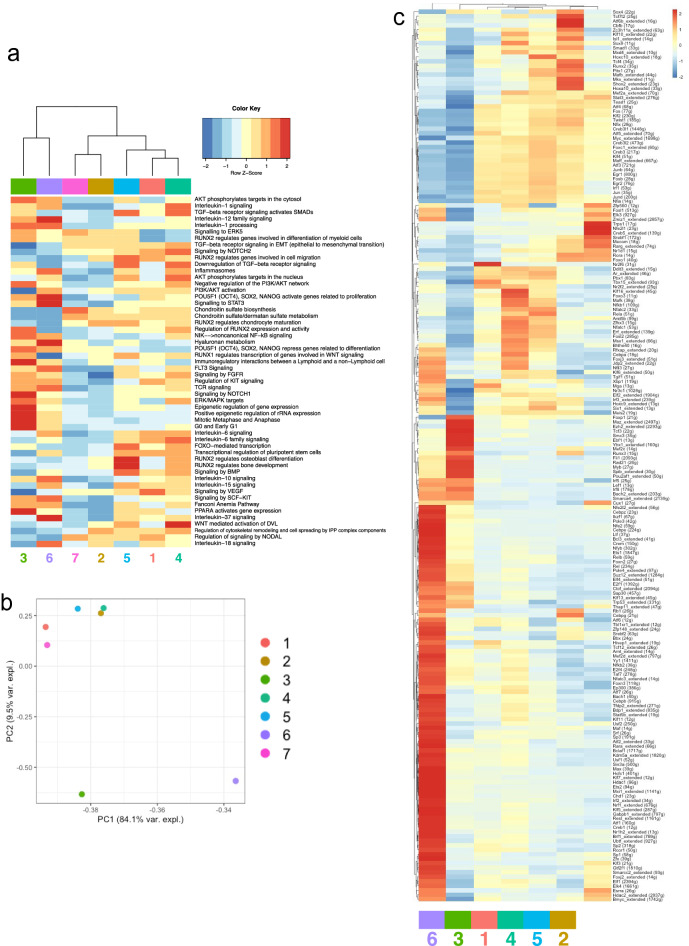
Down-stream analyses of scRNA-seq for the Pα-S fraction cells (n = 2367). Downstream analysis of cluster annotation in Fig. 1. (**a**) Heatmap of pathway enrichment comparing among clusters by ReactomeGSA. Comparison with the enriched pathways between each cluster. (**b**) Principle component analysis of enriched pathways in clusters. (**c**) Heatmap of regulatory transcriptional factors by SCENIC (color code as in Fig. 1a).

Cluster 1, 4 may have myeloid differentiation, cluster 2 may have hematopoiesis related, cluster 3 may have angiogenesis, cluster 5 is immune cell related, cluster 6 is chondrocyte related, cluster 7 may have osteoblast and bone development term were extracted (Fig. 2a). Principal component analysis from the GO terms characteristic of each cluster revealed the cluster 4 was similar to clusters 1, 2, and 5 (Fig. 2b). In addition, clusters 3 and 6 demonstrated a tendency to different enriched pathways from other clusters. These results considered that clusters 3 and 6 were progressed the differentiation. From a different gene expression profile in each cluster based on the cisTarget databases, the candidate transcription factor 'regulon' predicted from the upregulated gene set was scored for each cell. As a result, the expression of transcription factors was observed in characteristic of each cluster (Fig. 2c). Remarkably, the transcriptional factor expression highlighted distinct characteristics of clusters. Cluster 1 expressed Tbx15 that contributes to bone development; cluster 2 expressed Runx2 and Cbfb that regulate ossification process; cluster 3 expressed Runx3 and Mef2c that regulate the blood cell differentiation and play a role in skeletal muscle development; cluster 4 expressed Sox9 and Nfkb that regulate cartilage development; cluster 5 expressed Pbx1 and Mafb that play pivotal roles in regulating hematopoiesis; cluster 6 expressed Klf 3, 5, and 7 that regulate embyo development; cluster 7 expressed Stat3 and Trps1 that regulate chondrocyte proliferation and differentiation. These results indicate that cells isolated as MSCs in the previous research are a heterogeneous cell population where every member shows distinct gene expression properties.

### Identification of molecular markers enabling to isolate each of populations

**Figure 3:**
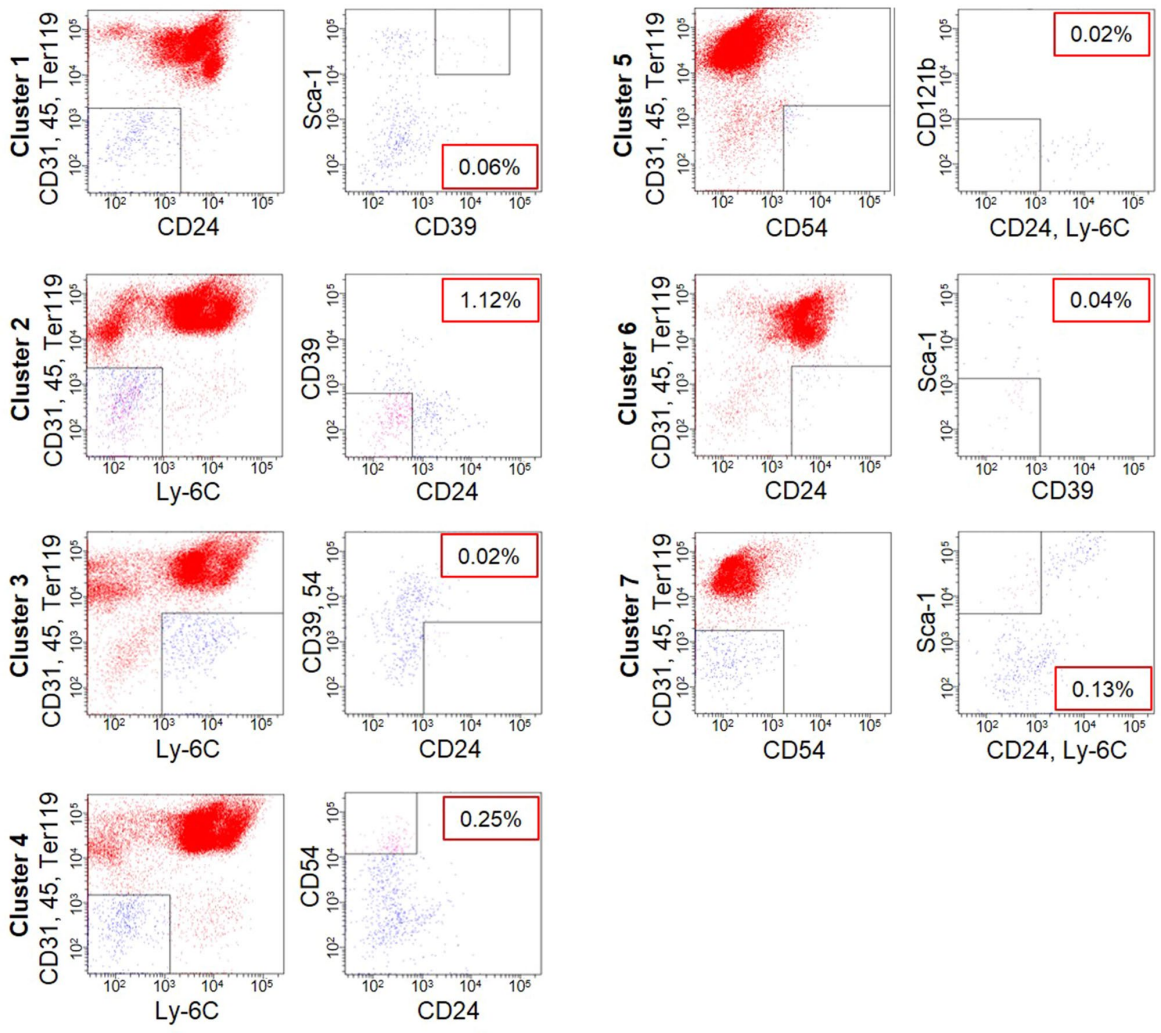
Isolation of each cluster using specific markers. Each cluster was isolated by using the surface antigen gene with large alteration in gene expression profiling by scRNA-seq of Pα-S.

To identify molecular markers enabling us to sort each of the identified populations, we listed candidate markers that are available for FACS sorting among the genes of which expressions showed a 1.5-fold or more increase or decrease in scRNA-seq (Fig. 1c, and Fig. S1, d). As a result, we found that most of the populations had common candidate marker genes CD24, 39, 54, Sca-1 and Ly-6C. When these were used together or different combination to isolate them, subpopulation could be separated at a rate of 0.02–1.12% of the total population (Fig. 3). Thus, the selection markers that we identified here were useful for isolation of individual subpopulations.

### Genetic character in each cluster by scRNA-seq and ATAC-seq

**Figure 4:**
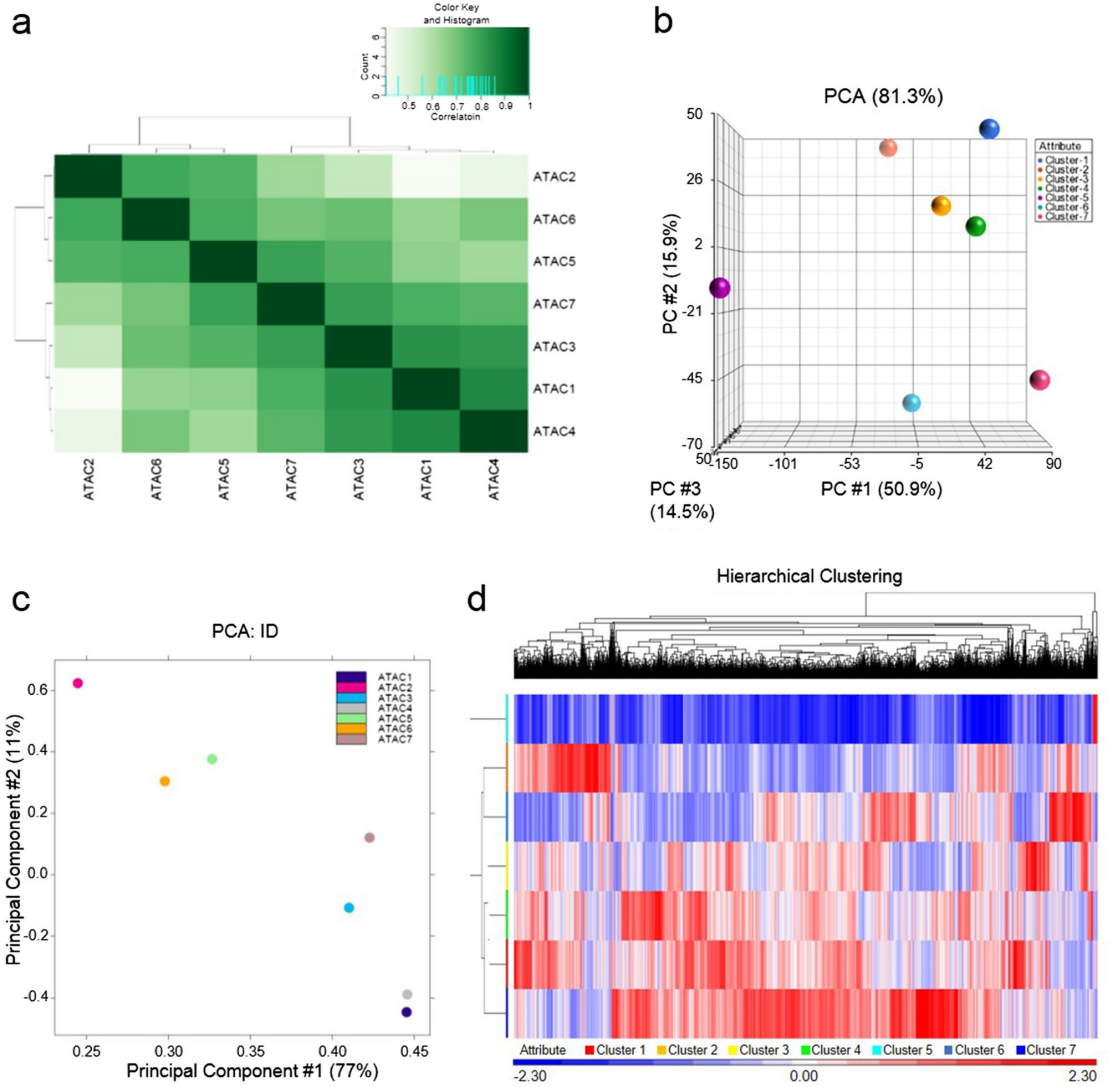
Identification of cluster-specific open chromatin regions and gene expression by ATAC-seq and RNA-seq analyses. (**a**) Clustering analysis for the peak intensity of each cluster in ATAC-seq. (**b**) PCA plots based on the peak intensity of ATAC-seq. (**c**) PCA plot analysis based on the gene expression profiles in RNA-seq analysis. (**d**) Clustering analysis based on gene expression in RNA-seq analysis.

To evaluate the genetic characteristics of the subpopulations identified by scRNA-seq, chromatin landscape and gene expression profiles were examined in each of the sorted population (clusters 1–7) by ATAC-seq and RNA-seq analysis, respectively. Based on the peak intensity of ATAC-seq, we conducted clustering analysis and made a PCA plot. The seven clusters were largely divided into two groups according to similarities of open chromatin signatures: group 1 comprised of clusters 1, 3, 4, and 7; group 2 comprised of clusters 2, 5, and 6. In the group 1, cluster 4 was most similar to cluster 1, and then similar to clusters 3 and 7 (Fig. 4a,b). When gene expression data obtained by RNA-seq were also subjected to clustering analysis and PCA plot analysis, we obtained similar results to the open chromatin signature; cluster 4 was most similar to cluster 1, and then similar to clusters 3 and 7, suggesting that the subpopulations identified by scRNA-seq were successfully sorted (Fig. 4c,d). These data indicate that each cluster is an independent population, along with genetic similarity between the clusters to some extent.

### Clusters possess the closest properties to stem cells

**Figure 5:**
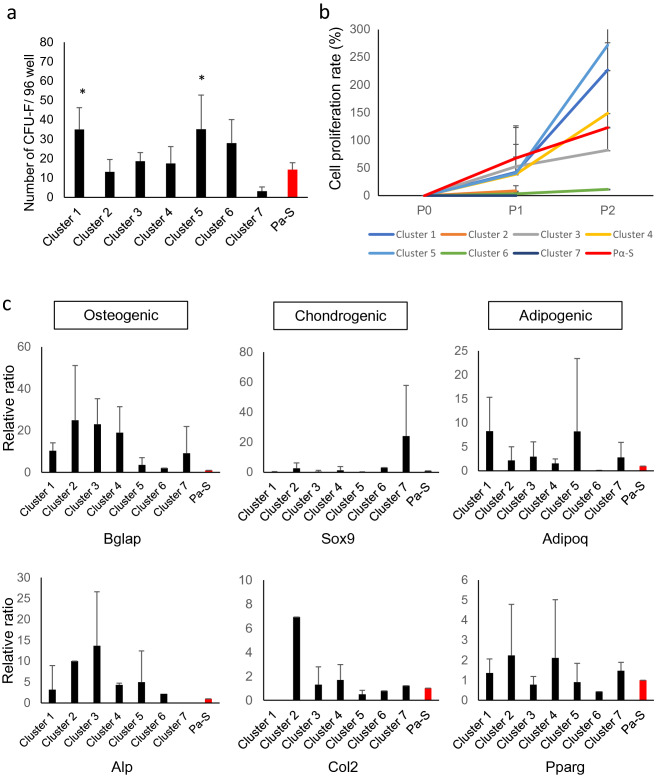
Stem cell properties of each cluster. (**a**) Graph showing bone marrow fibroblastic colony forming unit (CFU-F). *P < 0.01 versus Pα-S (n = 9). (**b**) Cell proliferation assay for each cluster. (**c**) Real Time PCR analysis for mesenchymal lineage marker expression in each cluster following 2-week treatment with mesenchymal differentiation media. **P < 0.05 versus Pα-S (n = 6).

To characterize each of the subpopulations functionally, we analyzed their CFU-F activities and differentiation potentials in vitro. Most of the subpopulations had the CFU-F activity as in the Pα-S group, while clusters 1 and 5 had significantly higher activity (Fig. 5a). Cluster 1 and 5 also showed vigorous proliferation in the cell proliferation assay. Cluster 3 and 4 proliferated at similar rate as PαS, while cluster 2, 6 and 7 showed much poorer proliferations (Fig. 5b). Furthermore, when these subpopulations were induced trilineage differentiation, each cluster showed peculiar pattern of the differentiation ability (Fig. 5c). However, there was no significant difference in the gene expression, because of high batch to batch variation. Cluster 1 and 5, which showed significantly higher CFU activity (Fig. 5a), tended to differentiate into osteogenic and adipogenic, but not chondrogenic linages. The cluster 4, 1 of the 2 clusters which showed comparable proliferation ability to PαS, showed tendency of higher expression of 5 genes out of 6 examined, and similar expression of Sox9 compared to PαS. Given that the differentiation state altered around cluster 4 in pseudotime analysis and cluster 4 showed more undifferentiated characteristics than other clusters (Fig. 1b), cluster 4 is likely to possess the closest properties to stem cells. Clusters 1 and 5 may also have similar properties. The number of cells is different between the Pα-s cell population, which is a bulk population, and each of subdivided clusters. In addition, it is predicted that the gene expression level per cell will also be different. We suppose that these features cause the high variation of the clusters.

### Functional significance in open chromatin regions by GREAT analysis

**Figure 6:**
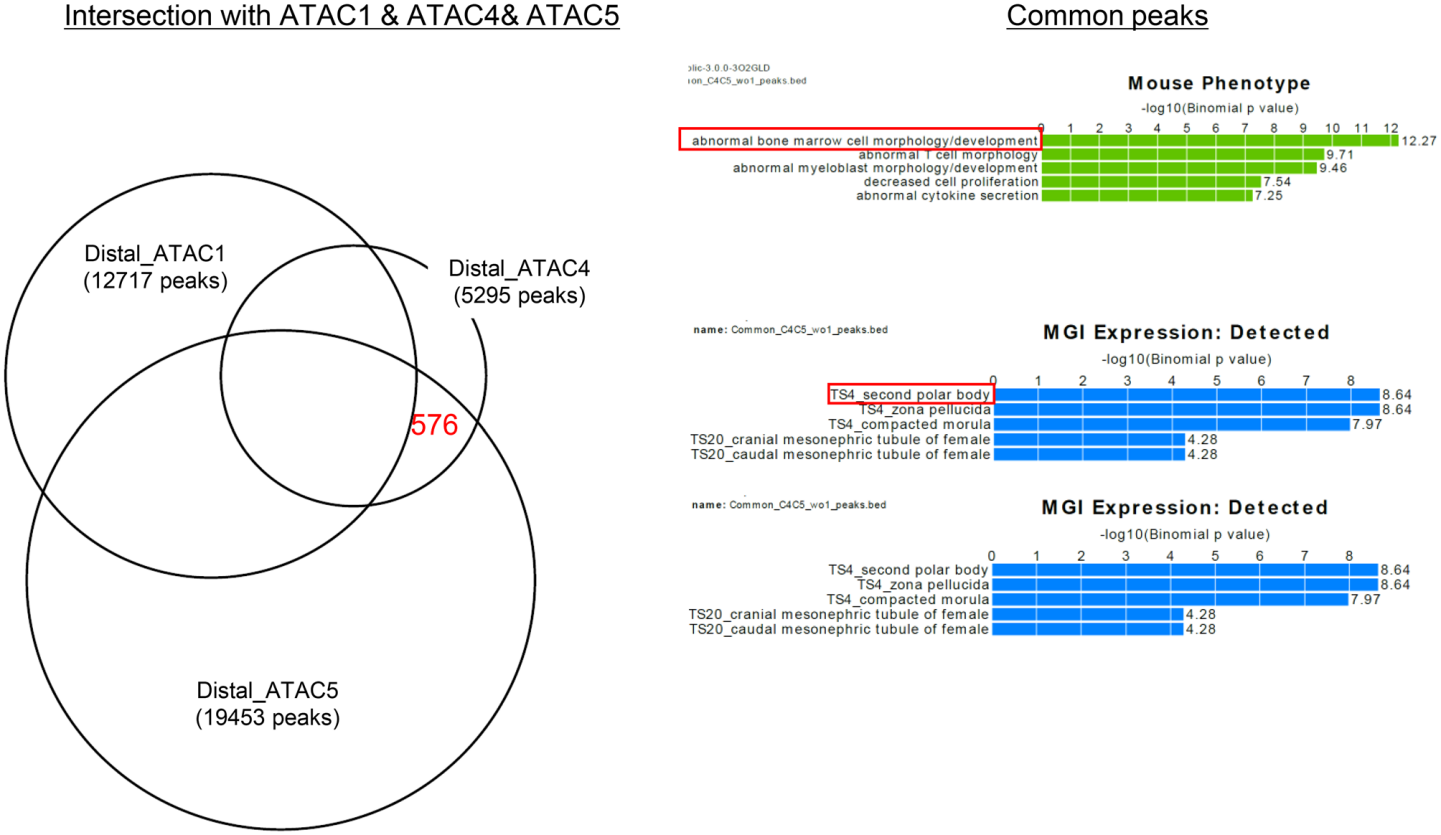
Analysis of open chromatin signals. Open chromatin regions common to clusters 4 and 5 and different from cluster 1 (left) were analyzed by GREAT analysis (right).

Given the stem cell-like characteristics of cluster 4, we next attempted to identify cluster 4-specific signatures. However, in comparison of ATAC-seq and RNA-seq data between all clusters, we found no cluster 4-specific open chromatin regions or expressed genes, although there is a similarity between open chromatin and the signature of gene expression (Fig. 4). We then focused on clusters 1, 4, and 5, of which properties were similar to stem cells, and compared their ATAC-seq peaks. Among the three clusters, cluster 1 and cluster 4 showed similar gene expression profile and open chromatin signature, while cluster 4 seemed to be closer to stem cells (Fig. S2, a). Therefore, at first, we focused on open chromatin regions that were present in clusters 4, but not in cluster 1, as these regions may be associated with stem cell property of cluster 4; we performed GREAT analysis on these regions to investigate their functional significance. The regions were associated with genes expressed in oocytes or pre-implantation embryos (secondary polar body, zona pellucida and compacted morula), indicating that the cluster 4-associated genes may represent immature stem cell property of the cluster (Fig. 6). Furthermore, we compared the open chromatin regions of cluster 4 and 5, because tri-linage differentiation assay also indicated more stem-cell like properties of cluster 4. Surprisingly, these 2 clusters shared the open chromatin regions indicating immature characteristics describe above (Fig. S2, b). From 576 open chromatin regions that were commonly present in clusters 4 and 5, but not in cluster 1 in the Venn diagram, we searched for open chromatin regions specific for clusters 4 that represented stem cell properties. However, it was difficult to precisely evaluate factors that represent stem cell properties such as immature and differentiation tropism, and that were expressed in a cluster-specific manner. These results indicate that subtle differences in signal variability and some key factors may affect the directional differentiation and maintenance of undifferentiated state, although detailed analysis is necessary to draw conclusion in the future.

## Discussion

Recently, genome structure has been known to play an important role in stem cell property. Previous studies for ES cells and iPS cells reported that genomic regions involved in differentiation-directed regulation undergoes epigenetic regulation by methylation or acetylation at a certain stage^29^. Regarding tissue stem cells, epigenomic analyses of HSC revealed that undifferentiated state was maintained by the polycomb complex protein^30^.

However, analysis of genome structure has not progressed for tissue stem cells such as MSCs because of the heterogeneity of cells collected using existing markers for MSCs. We hypothesized that if cells with different functions and characteristics existing in MSC could be individually separated, it is possible to elucidate the innate tissue stem cell, which is clearly different from previous studies. To verify the hypothesis, we took advantage of scRNA-seq. NGS technology has been dramatically improved in recent years, and it is now possible to perform detailed genetic analysis at the single-cell level for heterogeneous cell population including stem, progenitor and stroma cells, which were previously thought to be MSCs. Previous studies reported that the Pα-S population has some of the Lepr expression and properties common to CAR cells and Nestin-positive cells^23^. In addition, recent reports on bone marrow stem cell niche elucidated the gene expression profile of mesenchymal stromal cell populations present in bone marrows, including Lepr-positive cells and CAR cells^31–33^. Although Pa-S cells include a part of the Lepr-positive cell population, it is not mentioned in these studies. In this study, we suggest that Pα-S cells are an independent cell population as described before because Pα-S cell population was not observed in the expression of Cxcl12 and Lepr defined by CAR cells and Lepr-positive cells. Therefore, scRNA-seq, RNA-seq, and ATAC-seq data in this study are newly obtained datasets, which do not overlap with the previous studies.

Our scRNA-seq analysis revealed seven genetically distinct subpopulations in the mesenchymal stromal cell population. We then found genes that are mutually expressed in these cell populations by gene expression profiles and successfully separated cell populations individually by combination of previously established MSC markers CD24, 39, 54, and Sca-1. Therefore, in this study, we also confirm that these genes are useful markers to isolate subpopulations^5,14,34–42^.

Next, we hypothesized that the change of genome structure plays a key role for MSC properties, and we expected that inventive results could be obtained by ATAC-seq analysis in bone marrow-derived MSCs. This study provided the first molecular characterization and functional follow-up of these seven cell populations. Gene expression profiles in subdivided individual clusters and epigenome data obtained by our ATAC-seq analysis suggest potential mechanisms underlying the specification of each of these populations. Selective detection of the open chromatin structure on a genome-wide basis enables to elucidate the mechanism of gene regulation. As a result, the specificity of each cluster indicated an independent cell population from heterogenous cell population^43^.

Our in vitro studies suggest that clusters have functionally different properties, and bioinfomatic analysis on RNA-seq and ATAC-seq also confirmed the properties of each cluster. Therefore, we demonstrated profile of the genes by RNA-seq and ATAC-seq, and found that each cluster in MSCs has a distinct transcriptome and epigenome. Thus, our study directly addressed the question of how each cluster has independent features, identifying many known regulators that are potentially involved in the establishment of cell-type specific chromatin structures and gene expression programs.

ATAC-seq contained more information to identify cluster type-specific features. Indeed, our data showed that the expression of cell type-specific genes was well correlated with the chromatin accessibility of the corresponding promoters; the gene expression pattern in RNA-seq and open chromatin signature in ATAC-seq are in a good agreement. ATAC-seq can identify not only promoter regions, but also the open chromatin regions of intergenic regions, which presumably contain cell-type specific enhancer regions^44^. However, we failed to find cluster-specific open chromatin regions, as in stem cells characterized by differentiation directionality regulation and maintenance of immature. Given that higher order structure of the genome including the open chromatin status is set up prior to gene expression and not strictly specific for each cell types^45^, open chromatin is not necessarily associated with subpopulation-specific gene expression in MSCs. In addition, we could not confirm specific gene expression patterns that precisely characterize each of the clusters, suggesting that we focused on the stem cell properties.

In this study, we attempted to identify tissue stem cells in the bone marrow; our analysis did not detect previously reported ES- and iPS-like population in MSCs. However our GREAT analysis indicates that clusters plays an important role in organogenesis in early development. Thus, these results suggest that isolated clusters are available for treatment of tissues defects in bone and cartilage diseases. Further examination will require on whether or not the clusters have pulripotency.

## Methods

### Mice

All experimental procedures and protocols for the present experiments were approved by the ethics committee or institutional committee for animal research of the University of Tokyo and were performed in accordance with the Guide for the Care and Use of Laboratory Animals and the ARRIVE guidelines. C57BL/6JJcl mice (6–12 weeks old) were purchased from CLEA Japan, Inc. (Tokyo, Japan). C57BL/6-Tg(CAG-EGFP)10sb/J mice, transgenic mice that ubiquitously express EGFP under the control of the CAG promoter, 6–12 weeks of age were purchased from CHARLES RIVER LABORATORIES JAPAN. The mice were kept under specific pathogen–free conditions in our animal facility at the University of Tokyo.

### Preparation of BM cell suspension

Femurs, tibias and ilium were dissected and crushed with a scissors and a pestle. The crushed bones were gently washed once in HBSS+ (Hanks-balanced salt solution supplemented with 2% FBS, 10 mM Hepes, and 100 U/mL penicillin/ 0.1 mg/mL streptomycin solution), and the solution filtered through a cell strainer (BD Falcon) was discarded. The bone fragments were collected and incubated for 1 h at 37 °C in 20 mL of DMEM (Invitrogen) containing 0.2% collagenase (Wako Chemicals USA, Inc.), 10 mM Hepes and 100 U/mL penicillin/ 0.1 mg/mL streptomycin solution. The suspension was filtered with a cell strainer (BD Falcon) to remove debris and bone fragments, and collected by centrifugation at 400 g for 5 min at 4 °C. The pellet was immersed in 1 mL water (Sigma-Aldrich) for 5–10 s to burst the red blood cells, after which 1 mL of 2 × PBS (diluted the product from Sigma-Aldrich) containing 4% FBS was added, and the suspension was filtered through a cell strainer. These serial procedures are described in previous report^28^.

### RNA isolation and quantitative PCR

For RNA isolation from differentiated cells, live cells were collected in Tri-Reagent buffer (Sigma-Aldrich) and cell lysates were homogenized with 21G needle on ice. Reverse transcription was performed using the PrimeScript RT reagent kit (TaKaRa), following the manufacturer’s recommendations for a standard-yield reaction (15 min of amplification time). mRNA expression was normalized to Hprt1 (for the experiments represented in Fig. 5. Expression levels of mRNA were assessed by real-time PCR using the SYBR Green Master Mix (Thermo Fisher Scientific). For relative quantitation of gene expression, mouse-specific Gapdh and Hprt1 (Invitrogen) were used as internal controls. Gene expression assays show mean values over three biological replicates; the experiment was performed three times. All other PCR primer sequences are listed in the supplementary table.

### scRNA-seq

For the initial scRNA-seq experiment was performed using the Chromium Single Cell Gene Expression Solution (10× Genomics), following the manufacturer’s protocol. The MSC was isolated from ten 6-week-old mice (all males). Cells were stained with the anti-mouse antibodies CD31 PE-Cy7, CD45 PE-Cy7 and TER119 PE-Cy7 (Biolegend), Sca-1 PE (eBioscience) and PDGFR-α APC (eBioscience), and 30,000 Lin − Sca-1 + Pdgfr-α + were isolated using a BD Bioscience FACS Aria III Fusion. Cells were washed and resuspended in 250 μl FACS buffer (PBS, 2% FBS, 1 mM EDTA), targeting the required 1000 cells/μl concentration, accounting for a 10–20% loss. We pipetted 9.7 μl cell suspension (concentration of 913 cells/μl, ~ 8800 cells), targeting the recovery of ~ 5000 cells. Single-cell RNA-seq libraries were obtained following the 10× Genomics recommended protocol, using the reagents included in the Chromium Single Cell 3’ v2 Reagent Kit. Libraries were sequenced on the NextSeq 500 v2 (Illumina) instrument using 150 cycles (18 bp barcode + UMI, and 132-bp transcript 3’ end), obtaining ~ 5 × 10^8^ raw reads^46,47^.

### scRNA-seq data analysis

The 10× Genomics scRNA-seq data was processed using cellranger-2.1.0, default parameters and the mouse NCBI38/mm10 genome. Molecular counts were obtained for 2300 cells (filtered matrix), with 160,000 mean reads/cell, an average of 60.5% reads mapping to the transcriptome and 3000 median genes detected per cell. We also filtered outlier cells using the median absolute deviation from the median total library size (logarithmic scale) as well as total gene numbers (logarithmic scale), as implemented in scran36, using a cutoff of 3 (isOutlier, nmads = 3). Log (normalized expression) values were obtained using size factors per cell, estimated with scran. Genomic alignment rates and number of detected genes/cell suggested that our data were of high quality^48^. No cutoff of expressed gene features in each cell was performed (Supplemental Fig. 1a). Percentage of mitochondrial genes in all expressed genes was less than 8%, and its distribution on t-SNE plot is almost equal from the appearance (Supplemental Fig. S1b). This is why regressing out of mitochondrial genes was not performed.

Dimension reduction with t-SNE and grouping into 7 clusters were performed with cell Ranger^49^. Information of clusters and coordination on t-SNE plot was succeeded in downstream analyses. Trajectory analysis was performed with Monocle3^50^ onto Seurat object. Feature plots, a dot plot, and a heatmap of top 10 ranked differentially expressed genes were made by Seurat v4^51^. Pathway enrichment analysis was perfomed by ReactomeGSA (Griss J, Mol Cell Proteomics 2020), Enrichment of focused pathways was summarized in a heatmap, and trend of all the enriched pathways in each cluster was summarized with PCA plot. Regulatory gene network analysis was perfomed by SCENIC^52^. Relative activities of each regulatory transcriptional factor with its number of downstream regulated genes were summarized in a heatmap.

### FACS-based cell isolation of mouse cells

The isolated single-cell suspension was diluted to 0.75 or 1 × 107 cells/ml with FACS buffer (PBS with 2% FBS, 1 mM EDTA, 1% penicillin) and the following antibodies were added: anti-mouse CD31 PE-Cy7, anti-mouse CD45 PE-Cy7, anti-mouse TER119 PE-Cy7 (BioLegend) for selecting the Lin − population; anti-mouse Sca1-PE (eBioscience), anti-mouse CD140a APC (eBioscience) and anti-mouse CD24 PE, APC (eBiosciences) to enrich the Lin − population with ASPCs; anti-mouse CD39 PE, APC (eBioscience), anti-mouse CD54 PE, APC(eBioscience) with APC for separating populations negative and positive for the given marker.

The cells were incubated with the cocktail of antibodies on ice for 20 min protected from light, after which they were washed and stained with DAPI (Sigma-Aldrich) or propidium iodide (Molecular Probes) for assessing viability, and subjected to FACS using a Becton Dickinson FACSAria III Fusion sorter. Compensation measurements were performed for single stains using compensation beads (eBiosciences).

### Differentiation cultures

To induce adipocyte differentiation, subconfluent cells were cultured with 3 cycles of Adipogenic Induction Medium/ Adipogenic Maintenance Medium, with supplements from the Adipogenic Induction/Adipogenic Maintenance SingleQuot kit (Lonza). Each cycle consisted of feeding the subconfluent cells with the induction medium for 3 days, followed by 3 days of culture in the maintenance medium. After 14 days, the cells were harvested with TRI Reagent (Sigma-Aldrich). For chondrogenic differentiation, the 2 × 10^4^–2.5 × 10^5^ cells were seeded into a 15-mL conical tube. The tube was spun at 400 g for 5 min at room temperature, and the supernatant was aspirated. The cells were resuspended in 1 mL Differentiation Basal Medium Chondrogenic, with supplements from the Chondrogenic SingleQuot kit (Lonza), spun at 400×g for 5 min, and the medium was aspirated. The cells were resuspended in 1 mL of Differentiation Basal Medium Chondrogenic, supplemented with Chondrogenic SingleQuots kit, TGF-β3 (10 ng/mL; Lonza) and BMP-6 (500 ng/mL; R&D Systems), and spun at 150 g for 5 min at room temperature. The pellet was maintained with Differentiation Basal Medium changed every 3–4 days for 2 weeks. After 3 weeks, cell clumps were harvested with TRI Reagent. To induce osteocyte differentiation, subconfluent cells were cultured with Differentiation Basal Medium Osteogenic, supplemented with Osteogenic SingleQuots (Lonza) for 14 days. The cells were then harvested with TRI Reagent^15^.

### RNA-seq analysis

RNA-seq analysis was performed as described^53^. Briefly, 35,000 cells were collected and 30 ng of total RNA was subjected to analysis. The RNA libraries were constructed by mRNA sequencing via polyA selection kit. Sequencing was performed with Hiseq PE 2 × 150 (Illumina). The sequence reads were aligned and mapped using Partek Flow software v2.2 (Partek). The raw reads were first subjected to pre-alignment Quality Assurance and Quality Control (QA/QC). Any base below a Phred value of 20 was trimmed from either side of the read and reads shorter than 25 nt length were removed. The processed reads were aligned by Tophat2-2.0.8^54^ to mm9 reference genome. The mapping quality and coverage were checked by post-alignment QA/QC. Aligned reads were quantified and normalized as reads per kilo base length of transcript per million reads (RPKM). Heat map of hierarchical clustering and PCA plot were generated by Partek Genomics Suite. In the analysis, we used selected genes based on the following criteria: fold change > 2 and rpkm > 2 for the hierarchical clustering; rpkm > 2 for the PCA plot.

### ATAC-seq analysis

The ATAC-seq was performed as described^55^. Briefly, 50,000 cells were collected and lysed with lysis buffer containing 10 mM Tris–HCl, 10 mM NaCl, 3 mM MgCl2, and 0.1% IGEPAL CA-630. Tn5 transposase reaction using the Tagment DNA Enzyme 1 (TDE1) (Illumina) was carried out at 37 °C for 30 min. The reacted DNA was purified using QIAGEN MinElute PCR purification kit and amplified for 8–15 cycles to produce libraries for sequencing. The ATAC-seq libraries were sequenced on Illumina Hiseq X sequencer. The sequence reads were aligned to the mouse genome reference sequence mm9 by bowtie aligner^56^. Peak calling was performed by two-sample analysis on CisGenome software^57^ with a *P*-value cutoff of 10^–5^ comparing with the input control. Peaks were incorporated into further analysis displaying an FDR < 0.01. Correlation heat map and PCA plot were generated by DiffBind software in R with default setting. Peak intersection was performed by BEDTools-Version-2.16.2. For gene ontology analysis, GREAT GO analysis was performed utilizing the online Genomic Regions Enrichment of Annotations Tool (GREAT), version 3.0.1^58^ with default setting.

### Statistical analysis

The data are presented as the mean ± standard error of the mean (SEM) and each independent experiment shown was reproduced three to five times. The comparison between two conditions was done by unpaired t test. A one-way repeated-measures ANOVA was applied to identify significant differences among conditions or groups. When a significant difference was observed, the data were subjected to post hoc analysis. A *p* < 0.05 was considered significant.

## Supplementary Information

Supplementary Information 1.

Supplementary Information 2.

Supplementary Information 3.

Supplementary Caption.

## Data Availability

The scRNAseq, RNA-seq and ATAC-seq that support the findings of this study have been deposited in the GEO under accession code GSE171531. Data are further available in processed form for download and interactive browsing at https://www.ncbi.nlm.nih.gov/geo/query/acc.cgi?acc=GSE171531.
