# Supplementary figures and images for: Mesenchymal stromal cells in the bone marrow niche consist of multi-populations with distinct transcriptional and epigenetic properties
Source: Sci Rep. 2021 Aug 4;11:15811. doi: 10.1038/s41598-021-94186-5 (PMC8338933; doi:10.1038/s41598-021-94186-5)

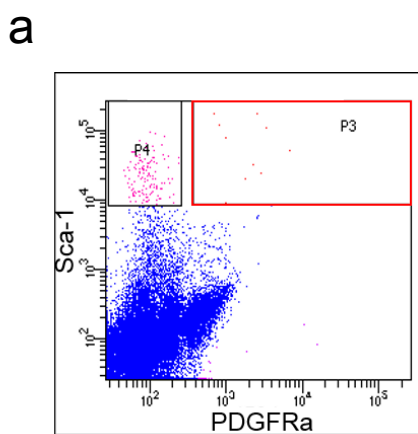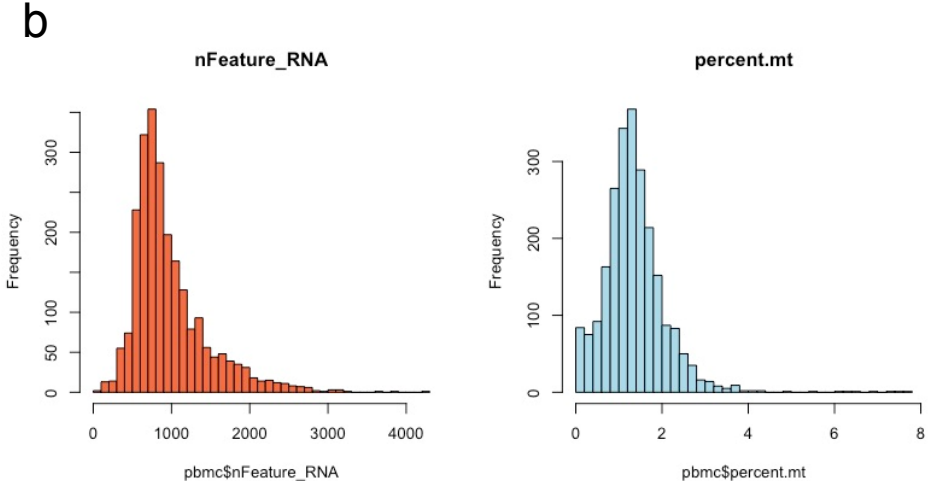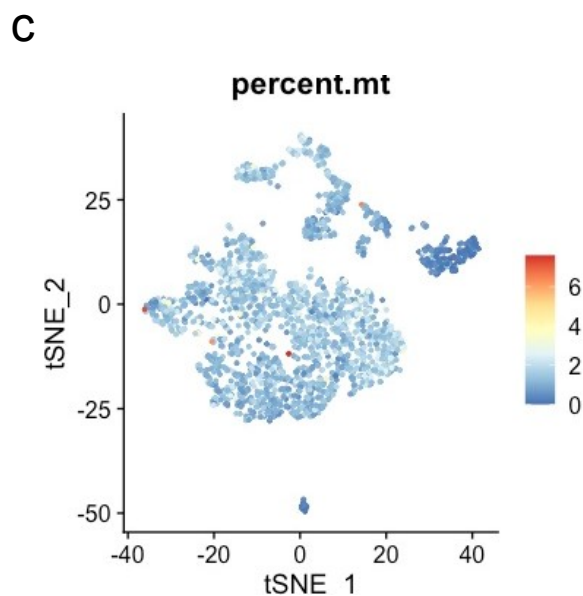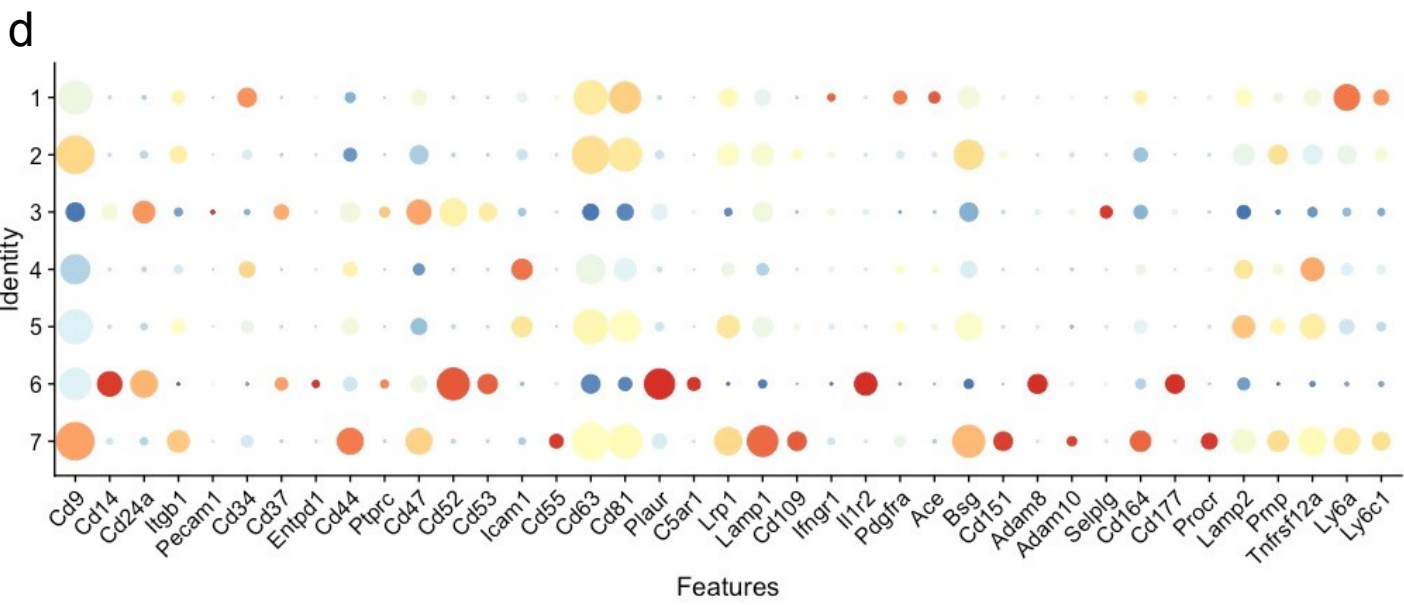

Supplement: Supplementary file 1 — Supplementary Information 1. [file 41598_2021_94186_MOESM1_ESM.pdf]

a

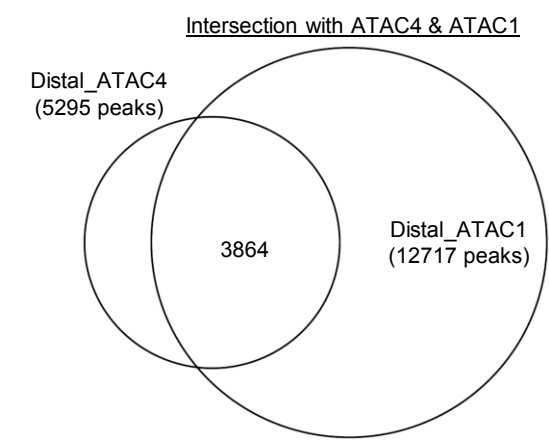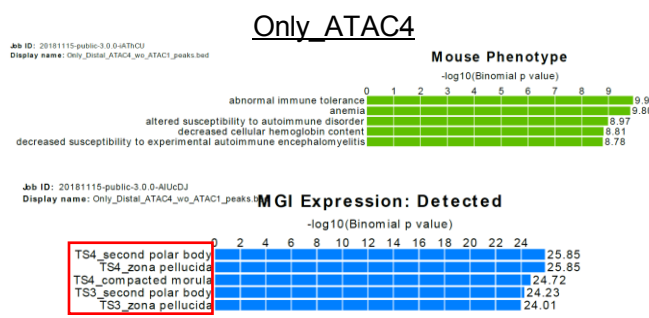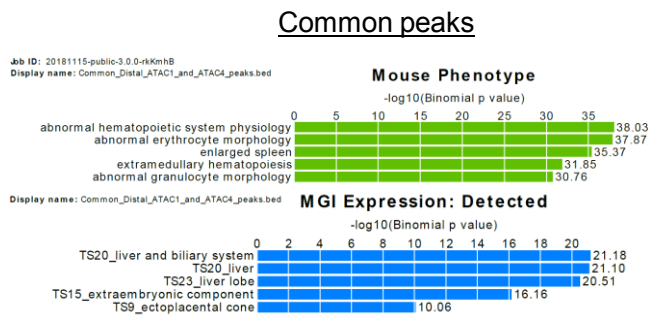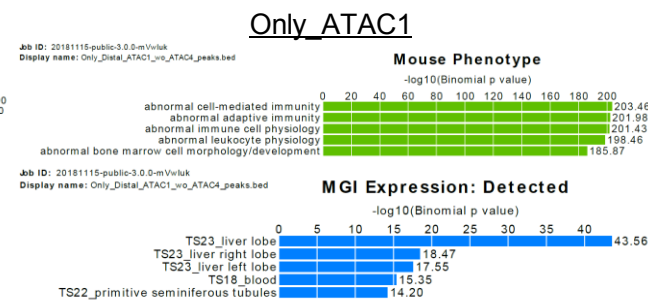

b

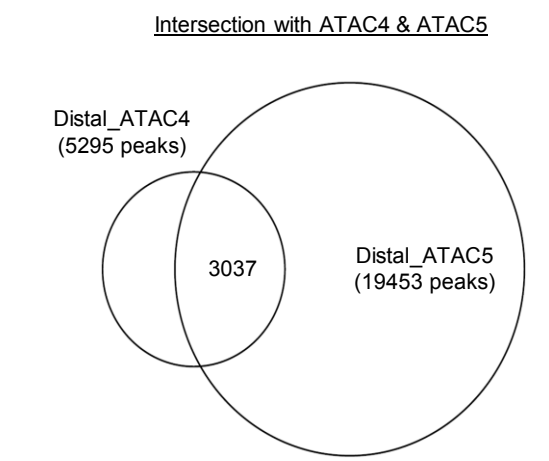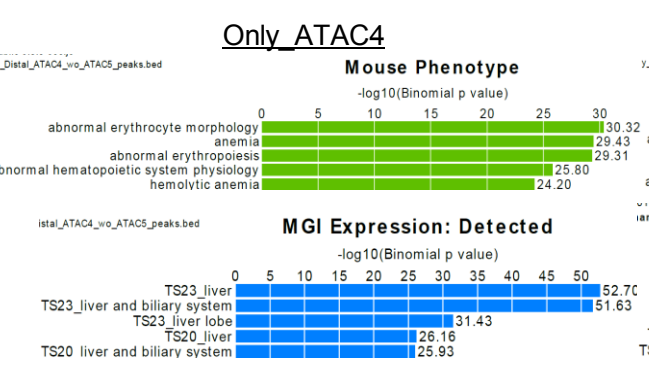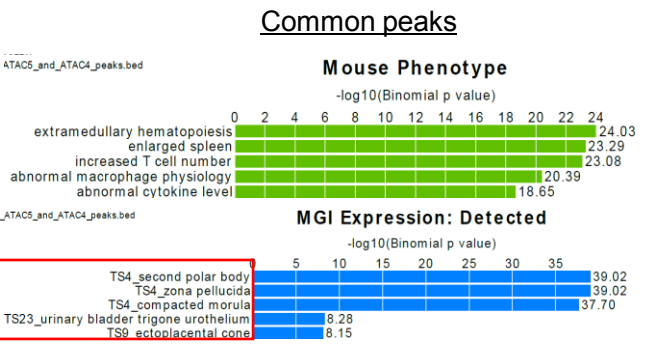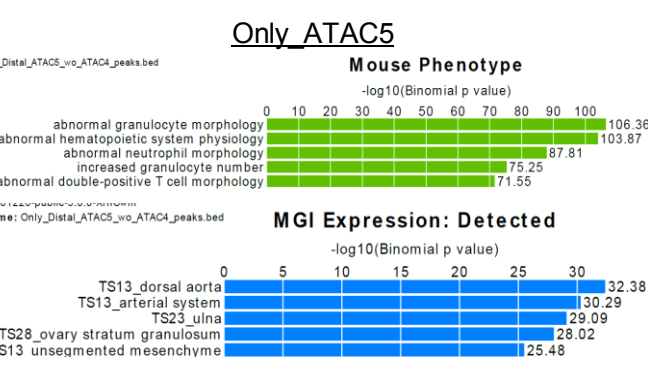

Supplement: Supplementary file 2 — Supplementary Information 2. [file 41598_2021_94186_MOESM2_ESM.pdf]
