# Supplementary material for: Mesenchymal stromal cells in the bone marrow niche consist of multi-populations with distinct transcriptional and epigenetic properties
Source: Sci Rep. 2021 Aug 4;11:15811. doi: 10.1038/s41598-021-94186-5 (PMC8338933; doi:10.1038/s41598-021-94186-5)
Supplement: Supplementary file 3 — Supplementary Information 3. [file 41598_2021_94186_MOESM3_ESM.docx]

**Supplementary Table**

Primers sequences for RT-PCR..

| **Gene name** |  | **Sequence** |
| --- | --- | --- |
| Gapdh | FW | 5'- CCACTAACATCAAATGGGGTGAGG -3' |
|  | RV | 5'- TACTTGGCAGGTTTCTCCAGGC -3' |
| Hprt | FW | 5'- TCAGTCAACGGGGGACATAAA -3' |
|  | RV | 5'- GGGGCTGTACTGCTTAACCAG -3' |
| Col2a1 | FW | 5'- TTGAGACAGCACGACGTGGAG -3' |
|  | RV | 5'- AGCCAFFTTGCCATCGCCATA -3' |
| Sox9 | FW | 5'- TCTCCTAATGCTATCTTCAAGGCG -3' |
|  | RV | 5'- TGCTCAGTTCACCGATGTCCAC -3' |
| Alp | FW | 5'- CACAATATCAAGGATATCGACGTGA -3' |
|  | RV | 5'- ACATCAGTTCTGTTCTTCGGGTACA -3' |
| Bglap | FW | 5'- GGGCAATAAGGTAGTGAACAG -3' |
|  | RV | 5'- GCAFCACAFFTCCTAAATAGT -3' |
| Adipoq | FW | 5'- TGTTCCTCTTAATCCTGCCCA -3' |
|  | RV | 5'- CCAACCTGCACAAGTTCCCTT -3' |
| Pparg | FW | 5'- ACCACTCGCATTCCTTTGAC -3' |
|  | RV | 5'- TGGGTCAGCTCTTGTGAATG -3' |
