# Supplementary material for: Mesenchymal stromal cells in the bone marrow niche consist of multi-populations with distinct transcriptional and epigenetic properties
Source: Sci Rep. 2021 Aug 4;11:15811. doi: 10.1038/s41598-021-94186-5 (PMC8338933; doi:10.1038/s41598-021-94186-5)
Supplement: Supplementary file 4 — Supplementary Caption. [file 41598_2021_94186_MOESM4_ESM.docx]

Mesenchymal stromal cells in the bone marrow niche consist of multi-populations with distinct transcriptional and epigenetic properties

Sanshiro Kanazawa, Hiroyuki, Okada, Hironori Hojo, Shinsuke Ohba, Junichi Iwata, Makoto Komura, Atsuhiko Hikita, Kazuto Hoshi

**Supplementary Figure 1**

Preprocess for scRNA-seq of the Pα-S fractions.

**Supplementary Figure 2**

A comparison between cluster 4 and 1 in open chromatin signals, and between cluster 4 and 5 in open chromatin signals.

**Supplementary Figure 1**

**Preprocess for scRNA-seq of the Pα-S fractions.** a) About 30,000 MSCs fractionated by PDGFRa, Sca-1 (Pα-S) were isolated from mouse bone marrow-derived mesenchymal cells. b) Histogram of number of feature genes and percentage of mitochondrial genes in single cell RNA-seq. No cutoff was set based on the distribution of Cell Ranger, and all 2,367 cells were analyzed. c) Distribution of percentage of mitochndrial genes on tSNE dimension reduction map. Regression out of mitochondrial genes was not performed, because the distribution of mitochondrial genes is not biased. d) Dot plot of gene expression of cell surface markers. As a result of a comprehensive study of cell markers, narrowed down to the cluster markers in Fig. 1c.

**Supplementary Figure 2**

**A comparison between cluster 4 and 1 in open chromatin signals, and between cluster 4 and 5 in open chromatin signals.** b) In the ATAC-seq analysis, focusing on regions common to clusters 4 and 1, genes listed as genes associated with terms obtained by GREAT analysis and their expression were listed. b) In the ATAC-seq analysis, focusing on regions common to clusters 4 and 5, genes listed as genes associated with terms obtained by GREAT analysis and their expression were listed.
